# Inflammation and Vascular Remodeling

**DOI:** 10.1155/2012/596796

**Published:** 2012-11-13

**Authors:** Ken-ichi Aihara, Masaki Mogi, Rei Shibata, David Bishop-Bailey, Muredach P. Reilly

**Affiliations:** ^1^Department of Medicine and Bioregulatory Sciences, Institute of Health Biosciences, The University of Tokushima Graduate School, 3-18-15 Kuramoto-cho, Tokushima 770-8503, Japan; ^2^Department of Molecular Cardiovascular Biology and Pharmacology, Ehime University Graduate School of Medicine, Shitsukawa, Ehime Tōon 791-0295, Japan; ^3^Department of Cardiology, Nagoya University Graduate School of Medicine, 65 Tsurumai, Showa-ku, Nagoya 466-8550, Japan; ^4^Centre of Translational Medicine and Therapeutics, William Harvey Research Institute, Barts and The London School of Medicine and Dentistry, Queen Mary University of London, London EC1M 6BQ, UK; ^5^Penn Cardiovascular Institute, Perelman School of Medicine, University of Pennsylvania, 11-136 TRC Building 421, 3400 Civic Center Boulevard, Philadelphia, PA 19104-6160, USA

## 1. Introduction

Cardio- and cerebrovascular diseases are the major causes of death worldwide. Accumulating evidence has revealed that inflammation plays a pivotal role in the progression of vascular remodeling and atherosclerosis leading to cardiovascular events. In fact, several inflammatory markers such as a high-sensitivity C-reactive protein have been shown to predict cardiovascular events. In addition, metabolic disorders, including hyperglycemia, hyperinsulinemia, and dyslipidemia, injure the vascular wall and contribute to the development of vascular remodeling. Most of these metabolic stimuli initially impair homeostasis of the cardiovascular system through inflammation with recruitment of leukocytes and increased secretion of adhesion molecules, chemoattractant cytokines, and proinflammatory cytokines from endothelial cells, vascular smooth muscle cells, fibroblasts, and macrophages. 

In this special issue, we invited front-line researchers and authors to submit original research and review articles that explore the interactions between inflammation and vascular remodeling. Finally, we were able to publish 6 review articles and 6 original research articles that provide pivotal evidence for understanding the pathophysiological roles of inflammation in vascular remodeling on a clinical basis and a molecular basis. The following papers will be useful for establishing treatment strategies to promote worldwide public health.

## 2. Review Articles

K. Ohshima et al. reviewed pathophysiological roles of peroxisome proliferator-activated receptor **γ** (PPAR-*γ*) in the process of development of atherosclerosis with vascular inflammation. They focused on novel insights into PPAR-*γ* activation and the immune system.

Since statins have not only cholesterol-lowering effects but also pleiotropic effects on the cardiovascular system, including anti-inflammatory and antioxidant effects and improvement of nitric oxide bioavailability, S. Yagi et al. focused on the effects of statins on cardiorenal syndrome on a clinical basis and a molecular basis.

Epoxyeicosatrienoic acids (EETs) have been shown to exert various biological effects on the vasculature including relaxation of vascular tone, cellular proliferation, and angiogenesis. S. J. Thomson et al. introduced the accumulating evidence of the anti-inflammatory effects of EETs in the cardiovascular system.

K. Yoshimura et al. previously reported the usefulness of c-Jun N-terminal kinase, a proinflammatory signaling molecule, as a nonsurgical therapeutic target for abdominal aortic aneurysm (AAA). Therefore, in their paper, they focused on recent advances in pharmacological intervention against the development of AAA. 

Recent genome-wide association studies have shown that ABO blood groups are associated with various disease phenotypes, particularly cardiovascular diseases. H. Zhang et al. reviewed the clinical importance of ABO blood groups as a locus for thrombosis, myocardial infarction, and multiple cardiovascular risk biomarkers.

MicroRNAs (miRNAs) are small noncoding RNAs of 18–22 nucleotides in length that regulate gene expression post-transcriptionally. In the past decade, miRNAs have been revealed to be novel regulators of vascular inflammation. In their paper, M. Yamakuchi overviewed the roles of miRNAs during vascular inflammation.

## 3. Original Research Articles

Oil thermoxidation generates oxidative free radicals that induce vascular inflammation. C. H. Ng et al. showed that prolonged consumption of repeatedly heated palm oil causes acceleration of vascular remodeling and hypertension. They concluded that the adverse phenotype is induced by increased VCAM-1 expression on endothelial cells. 

A. Sakamoto et al. demonstrated that both ephrin-B1 and its cognate receptor EphB2 exhibited higher expression levels in human abdominal aortic aneurysms, and that they are expressed in macrophages, T lymphocytes and endothelial cells. They also found that membrane-bound ephrin-B1 and EphB2 inhibited chemotaxis of human peripheral blood mononuclear cells.

Y. Izumiya et al. revealed that C-type natriuretic peptide (CNP) attenuated angiotensin-II (Ang-II) induced cardiac hypertrophy, fibrosis, and contractile dysfunction through reduction in cardiac superoxide production. Their results may partly be explained by the fact that CNP reduces cardiac expression of NOX4, a subunit of NADPH oxidase.

R. L. Maurice et al. introduce an imaging-based biomarker (ImBioMark) approach for assessing *in vivo *arterial stiffness in rat models. They also presented preliminary data on the potential of ImBioMark to evaluate post-Kawasaki disease vasculitis in pediatric patients.

Since *Candida albicans* water-soluble fraction (CAWS) has a strong induction potency for murine vasculitis and shows acute lethal toxicity, N. Hirata et al. proposed that CAWS-induced arteritis is an easy and unique heart failure model without the requirement of specific experimental techniques.

The laser microdissection (LMD) method has been used for collecting target cells from the microscopic regions for malignant tumor studies. In their paper, A. Ikeda et al. showed that the LMD method enables separate collection of muscular and vascular samples and selective evaluation of gene expression in individual myocardial tissues.

*Ken-ichi Aihara* *Ken-ichi Aihara* *Masaki Mogi* *Masaki Mogi* *Rei Shibata* *Rei Shibata* *David Bishop-Bailey* *David Bishop-Bailey* *Muredach P. Reilly* *Muredach P. Reilly*

